# THE ECONOMIC VALUE OF FAMILY CAREGIVING: NEW NATIONAL ESTIMATES AND STATE VARIATION

**DOI:** 10.1093/geroni/igz038.2742

**Published:** 2019-11-08

**Authors:** Ari Houser, Ari Houser

**Affiliations:** AARP Public Policy Institute, Washington, District of Columbia, United States

## Abstract

The economic value of family caregiving, by any measure, dwarfs actual expenditures on formal long-term services and supports (LTSS). This presentation discusses new estimates of the number of caregivers, intensity of caregiving, and the total economic value of family caregiving in 2017 in the United States, and in every state, the District of Columbia, Puerto Rico, and the Virgin Islands, based on a meta-analysis of recent nationally representative surveys of family caregivers. Previous analyses of this type have found that the total economic value of family caregiving has increased steadily from $350 billion in 2005 to $470 billion in 2013. State variation in the incidence, intensity, and economic value of caregiving will be discussed, and key predictors of this variation will be identified.

